# Repeated otilonium bromide administration prevents neurotransmitter changes in colon of rats underwent to wrap restraint stress

**DOI:** 10.1111/jcmm.13016

**Published:** 2016-11-20

**Authors:** Chiara Traini, Stefano Evangelista, Vincent Girod, Maria Simonetta Faussone‐Pellegrini, Maria Giuliana Vannucchi

**Affiliations:** ^1^ Department of Experimental and Clinical MedicineResearch Unit of Histology and EmbryologyFlorenceItaly; ^2^ Ricerche SpAPreclinical Development Dept.FlorenceItaly; ^3^ SyncrosomeMarseilleFrance

**Keywords:** corticotrophin releasing factor receptors, immunohistochemistry, nerve structures, inflammation, irritable bowel syndrome, otilonium bromide, smooth muscle cells, rat colon

## Abstract

Otilonium bromide (OB) is a spasmolytic drug successfully used for the treatment of irritable bowel syndrome (IBS). Its efficacy has been attributed to the block of L‐ and T‐type Ca^2+^ channels and muscarinic and tachykinin receptors in the smooth muscle. Furthermore, in healthy rats, repeated OB administration modified neurotransmitter expression and function suggesting other mechanisms of action. On this basis, we investigated whether repeated OB treatment prevented the functional and neurochemical changes observed in the colon of rats underwent to wrap restrain stress (WRS) a psychosocial stressor considered suitable to reproduce the main IBS signs and symptoms. In control, WRS and OB/WRS rats functional parameters were measured *in vivo* and morphological investigations were done *ex vivo* in the colon. The results showed that OB counteracts most of the neurotransmitters changes caused by WRS. In particular, the drug prevents the decrease in SP‐, NK1r‐, nNOS‐, VIP‐, and S100β‐immunoreactivity (IR) and the increase in CGRP‐, and CRF1r‐IR. On the contrary, OB does not affect the increase in CRF2r‐IR neurons observed in WRS rats and does not interfere with the mild mucosal inflammation due to WRS. Finally, OB *per se* increases the Mr2 expression in the muscle wall and decreases the number of the myenteric ChAT‐IR neurons. Functional findings show a significantly reduction in the number of spontaneous abdominal contraction in OB treated rats. The ability of OB to block L‐type Ca^2+^ channels, also expressed by enteric neurons, might represent a possible mechanism through which OB exerts its actions.

## Introduction

Otilonium bromide (OB) – also known by its IUPAC chemical name *N*,*N*‐diethyl‐*N*‐methyl‐2‐(4‐[2‐(octyloxy)benzamido]benzoyloxy)ethanaminium bromide – is prescribed in Europe, Asia and South America as an anti‐spasmodic for the treatment of irritable bowel syndrome (IBS) 1. This drug contains a charged quaternary ammonium that favours its accumulation within the gastrointestinal wall 2. The OB spasmolytic activity is mainly because of the interference of calcium influx through L‐type Ca^2+^ channels and the mobilization of cellular calcium required for smooth muscle contraction, thereby limiting excessive intestinal contractility. Otilonium bromide also inhibits T‐type Ca^2+^ channels and muscarinic and tachykinin receptors (NK1r and NK2r) on smooth muscle and primary afferent neurons that may have the joint effect of reducing motility and abdominal pain 3. Indeed, in strips of human colon, exposure to OB, either alone or in the presence of the tachykinin agonist, caused a concentration‐dependent reduction in the number of the cells that internalized the receptor NK2r 4.

A recent clinical trial has confirmed the OB efficacy in IBS patients and has shown, for the first time, the long‐term beneficial effect of the drug during the follow‐up period in a group of patients previously treated with OB as compared to placebo group 5. These findings suggest that OB given in a long‐term way is able to preserve the clinical beneficial effects beyond its interruption.

Several experiments have been done in rats repeatedly treated with OB to ascertain its cellular targets and mechanisms of action. The results obtained demonstrated: in the smooth muscle cells, morphological changes in the structures involved in calcium handling and storage (caveolae, smooth endoplasmic reticulum, mitochondria) and in the NK1r distribution and Mr2 expression; in the enteric neurons, immunohistochemical and functional changes in the nNOS/SP/NK1r mediated signalling circuit 6, 7, 8.

Colonic biopsies from IBS patients show an increase in activated lymphocytes, mast cells, and neutrophilic and eosinophilic granulocytes in the mucosa suggestive for a local inflammation 9, 10. Moreover, an increase in the transient receptor potential vanilloid 1 no‐selective ionic channel and SP expression has been reported and related to pain 11. A main role in the aetiopathogenesis of IBS has been attributed to a psychosocial stress and animal models based on this hypothesis and reproducing some of the most common IBS symptoms have been developed. We recently demonstrated in rats underwent to the wrap restrain stress (WRS) the development of an increased colonic activity and faecal excretion and, as in IBS patients, a mild inflammation in the mucosa 12. Interestingly, we also found important changes in glial cells and in excitatory and inhibitory neurotransmitters in the entire colonic wall, and an increase in CRF1r expressing myenteric neurons.

The peculiar features of the WRS model make it particularly suitable to investigate the ability of OB to prevent or attenuate the appearance and evolution of the colonic alterations induced by stress. Therefore, this study was aimed to investigate whether a repeated treatment with OB prevented the functional and immunohistochemical changes characterizing the WRS rats.

## Materials and methods

### Animals

Male Wistar rats (*n* = 71) from Janvier Labs (Saint‐Berthevin, France) weighing around 250–350 g on the day of experiments were used for this study and housed at Syncrosome Laboratories (Marseille, France). The acclimatization of the animals lasted at least 5 days. They had free access to food and drinking water *ad libitum*. The experimental procedures were carried out in accordance to European guidelines for the care and use of laboratory animals (Directive 2010/63/UE) and were approved by the French Ethical Committee C2EA‐/71, of which Syncrosome is a member. Every effort was done to maintain animal's health, safety and welfare, minimize suffering and reduce the number of animals used in the experiments. Furthermore, Syncrosome laboratories and its animal housing facilities are accredited by French Authorities and audited every year by French Department of Veterinary Service.

### Study plan

**Figure 1:**
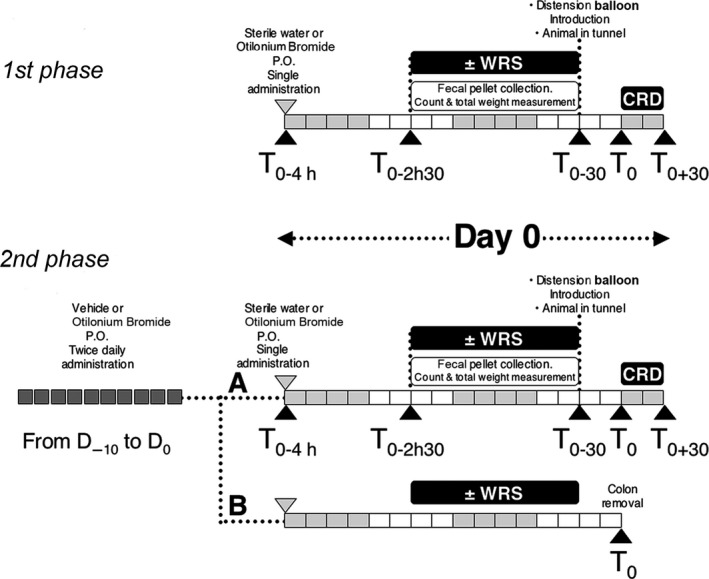
Study plan. First phase: OB was given at 2 mg/kg and at 20 mg/kg, 5 ml/kg, 4 hrs before colorectal distension (CRD). WRS was induced for 2 hrs. Second phase: OB given at 20 mg/kg, 5 ml/kg, was administered for 10 days before inducing WRS.

The study was divided into two consecutive phases. The first phase was carried out to better define the OB dose to be used in the second one, consisting in the main session of experiments. First phase: OB was given 4 hrs before colorectal distension (CRD) and abdominal contraction (AC) recordings at doses of 2 or 20 mg/kg p.o. (Fig. 1 and Table S1). These doses and time were chosen on the basis of previous experiments 6, 7 and of pharmacokinetic properties of the drug 2. Second phase: The chosen OB dose of 20 mg/kg was administered for 10 days and on the day 11, the rats received the last dose 4 hrs before undergoing to CRD (Fig. 1 and Table S1). In both phases, rats were subjected to WRS for 2 hrs, starting at 1.5 hrs after the last OB administration. For the total number of rats used for each phase, see Table S1.

### OB preparation and administration

The drug was dissolved in water at final concentrations of 0.4 and 4 mg/ml and a total volume of 5 ml/kg was administered to reach the 2 or 20 mg/kg dose respectively. The controls received an equal volume of water. Otilonium bromide was given p.o. For the first phase, OB was administered once at Day 0 (D_0_) 4 hrs before CRD test; for the second phase OB was administered twice daily from D_−10_ to D_0_ and the last dose on D_0_ 4 hrs before CRD test (Fig. 1 and Table S1).

### Application of WRS and functional evaluations

The application of WRS consists in the block of the upper forelimbs and thoracic trunk for 2 hrs. The timing and the procedure of WRS application was described in detail in Data S1. Faecal pellet collection and ACs following colorectal distention by the insertion of a balloon were chosen as functional parameters. The details about the timing of functional evaluations and methods were explained in Data S1.

### Tissue sampling

As depicted on Figure 1 and Table S1, the animals that did not undergo to CRD were killed at T_0_ with pentobarbital and the colon was removed. In particular, three full‐thickness samples (length: 1 cm/each) of the ascending colon (starting 1 cm far from the ileo‐caecal junction) were taken from each experimental group and randomly assigned to the different procedures described below. For paraffin embedded tissue, the sample was fixed in 4% paraformaldehyde in 0.1 M phosphate buffered saline (PBS, pH 7.4) over night (ON) at 4°C, dehydrated in graded ethanol series, cleared in xylene and embedded in paraffin. Then, the sections (5 μm thick) were cut using a rotary microtome (MR2; Boeckeler Instruments Inc., Tucson, AZ, USA) and collected on positively charged slides. For frozen tissue, a second sample after fixation was cryoprotected with 30% sucrose in PBS ON at 4°C, embedded in Killik cryostat medium (Bio‐Optica, Milan, Italy) and frozen at −80°C. Transverse cryosections (8 μm thick) were cut using a cryostat (Leica Microsystem CM 1950, Wetzlar, Germany) and collected on positively charged slides. For western blot analysis, a third sample was quickly frozen in liquid nitrogen and stored at −80°C. To evaluate the tissue organization, either paraffin embedded or frozen sections were stained with haematoxylin and eosin.

### Immunohistochemistry

The paraffin embedded sections were deparaffinized and rehydrated as usual and for antigen retrieval boiled 10 min. in sodium citrate buffer (10 mM, pH 6.0; Bio‐Optica) or treated for 20 min. at 90–92°C in Tris buffer (10 mM) with ethylenediaminetetraacetic acid (1 mM, pH 9.0), as appropriate, followed by cooling to room temperature (RT).

For fluorescence microscope examination, the frozen and the deparaffinized sections were washed in PBS and blocked for 20 min. at RT with 1.5% bovine serum albumin (BSA; Applichem, Darmstad, Germany) in PBS, plus 0.5% Triton (Sigma‐Aldrich, St. Louis, MO, USA) for the frozen ones. The primary antibodies diluted in PBS were applied ON at 4°C. The day after, the sections were washed in PBS and incubated for 2 hrs at RT in the dark with appropriate fluorochrome‐conjugated (AlexaFluor 488‐ or 568‐conjugated) secondary antibodies (goat anti‐rabbit, goat anti‐mouse, donkey anti‐goat, or goat anti‐guinea pig, Invitrogen, San Diego, CA, USA) diluted 1:333 in PBS. The sections were washed in PBS and mounted in an aqueous medium (Immuno‐Mount; Thermo scientific, Rockford, IL, USA) with or without 4′,6‐diamidino‐2‐phenylindole (Sigma‐Aldrich). The immunoreaction products were observed under an epifluorescence Zeiss Axioskop microscope (Zeiss, Oberkochen, Germany) using 488‐, 568‐ and 370‐nm excitation wavelength for the green, red and blue fluorescent labels, respectively, and the fluorescence images were captured using a Leica DFC310 FX 1.4‐megapixel digital camera, equipped with the Leica software application suite LAS V3.8 (Leica Microsystems, Mannheim, Germany).

For light microscope examination, after deparaffinization, rehydration and antigen retrieval phase as reported, the sections were treated with 3% H_2_O_2_ in PBS for 5 min. to block endogenous peroxidase activity. After washing, the sections were incubated with 1.5% BSA in PBS for 20 min. at RT. The anti‐S100β primary antibody was applied ON at 4°C. The day after, the sections were incubated with the biotinylated secondary antibody diluted 1:300 (goat anti‐mouse; Jackson ImmunoResearch, West Grove, PA, USA) in PBS for 2 hrs at RT. Subsequently, the sections were washed, treated with ABC Reagent (Vectastain ABC/Elite Kit; Vector Laboratories, Burlingame, CA, USA) for 20 min. and developed with 3,3′‐diaminobenzidine (DAB; Sigma‐Aldrich), as chromogen. Sections were counter‐stained with haematoxylin. After washing in PBS, the sections were mounted in an aqueous medium (Sigma‐Aldrich) and observed under the Microstar IV light microscope (Reichert Technologies, Reichert Inc, Depew, USA). For CRF1r staining, the procedure described by 13, 14 has been followed. Endogenous peroxidase activity was blocked by incubation for 30 min. with 0.3% H_2_O_2_ in PBS and nonspecific reaction was blocked by incubation in 3% normal donkey serum (60 mg/ml; Jackson ImmunoResearch) for 30 min. at RT. Sections were incubated with anti‐CRF1r in PBS 0.3% Triton X‐100 and the day after with the secondary antibody (rabbit anti‐goat; Jackson ImmunoResearch). Finally, these sections were processed for avidin‐biotin‐peroxidase procedure using DAB and observed under the light microscope.

Negative controls were performed omitting the primary antibody or using the specific blocking peptide to exclude the presence of nonspecific staining.

### Western blot

The procedure followed foreseen the protein content extraction from full‐thickness samples of ascending colon. After spectrophotometrically protein quantification, samples and appropriate molecular‐weight markers were resolved by standard electrophoresis and blotted onto polyvinylidene fluoride microporous membrane. After the incubation with primary and peroxidase‐conjugated secondary antibodies, the immunoreaction products were revealed by chemiluminescence reagent. To normalize the values of the antibody, the runs were also immunostained with anti‐β‐actin, assumed as housekeeping protein. The details about western blot procedures were explained in Data S1.

Information on primary antibody sources and used concentrations for fluorescence, light microscope examination and WB analysis is shown in Table S2.

### Quantitative and statistical analysis

The faecal pellets and AC number was quantified and expressed as number ± S.E.M. A quantitative analysis of the immunoreactive (IR) structures was done by the acquisition of digitized images of the labelling (1 section/animal; 5 animals/group) using 20× or 40× objectives, as appropriate; attention was made to avoid overlapping between the adjacent portions. Each image was analysed using ImageJ (NIH, Bethesda, ML, USA) to evaluate the intensity of labelling and to quantify the area of the IR structures. The photographs’ threshold values were set to analyse the structures of interest exclusively. The labelling was converted to a binary image and the number of pixels and the IR intensity were measured. The results are expressed as optical density ± S.E.M. The quantitative analysis of the number of IR neurons or inflammatory cells was done by two observers, blind to each other, along the entire section (three sections/animal; five animals/group) and expressed as number ± S.E.M. The quantitative analysis of western blot‐IR bands was performed by computer‐assisted densitometry by using the QuantityOne analysis software (Bio‐Rad, Hercules, CA, USA) with each band corresponding to a single specimen. The results for the protein of interest were normalized to the corresponding β‐actin band.

Statistical analysis was performed by one‐way anova followed by Newman–Keuls post‐test to compare the three groups. Differences were considered significant when *P* < 0.05.

## Results

### Functional recordings

#### First phase

A statistically significant increase (*P* < 0.05) in the weight and number of faecal pellets was found in rats subjected to WRS as compared to controls (2869 ± 416 *versus* 986 ± 219 mg and 10.63 ± 1.97 *versus* 5.88 ± 1.27 respectively). Acute administration of OB (2 and 20 mg/kg p.o.) did not affect these parameters (data not shown). AC numbers of rats subjected to WRS was increased only at 1.2 ml distension as compared to controls (21.00 ± 3.09 *versus* 14.43 ± 2.62). This increase (35% from controls) was reduced by 20 mg/kg p.o. OB at 14.63 ± 1.89 and by 2 mg/kg p.o. OB at 17.88 ± 2.97. On the basis of these results, the dose of 20 mg/kg OB was chosen for the next experiments.

#### Second phase

Number and weight of faecal pellets showed a statistically significant increase (*P* < 0.05) in the WRS group as compared to control one (10.0 ± 1.64 *versus* 7.25 ± 1.10 and 2724 ± 364 *versus* 1146 ± 151 mg respectively). The repeated treatment with 20 mg/kg of OB was unable to affect these parameters (values in number and weight of faecal pellets were: 10.25 ± 1.22 and 2327 ± 277 mg). Increase in distension volume produced an increase in AC in all groups. Controls: 3.75 ± 1.39, 7.75 ± 0.86, 13.25 ± 1.79, 21.75 ± 2.30; WRS rats: 4.13 ± 1.11, 5.88 ± 1.57, 18.13 ± 1.92, 29.00 ± 2.00; OB/WRS rats: 1.75 ± 0.56, 6.50 ± 1.73, 17.38 ± 1.61, 25.25 ± 3.52 at 0.0, 0.4, 0.8 and 1.2 ml of volume distension, respectively. The increase was statistically significant (*P* < 0.05) as compared to 0 volume distension at 0.4, 0.8 and 1.2 ml of volume distension in controls and at 0.8 and 1.2 ml in WRS and OB treated animals. Comparison between controls and WRS rats showed a statistically significant increase (*P* < 0.05) in the latter group at 0.8 and 1.2 ml of volume distension. OB/WRS did not show any significant difference in the number of AC at any volume of distension examined as compared to WRS rats. However, at variance with WRS rats, the number of AC in OB treated rats at 1.2 ml of volume distention was not significantly different from controls. To note, in the absence of volume distension, OB/WRS group showed a statistically significant decrease (*P* < 0.05) in number of AC (1.75 ± 0.56) *versus* WRS (4.13 ± 1.11) and control rats (3.75 ± 1.39).

### Morphological findings

Histological evaluation of the colonic wall with haematoxylin and eosin showed a substantial integrity of mucosa, submucosa and muscle wall in all groups of animals. Similarly, the labelling with the pan‐neuronal marker PGP9.5 showed no changes among the groups in the total number of neurons either in the myenteric or submucous plexus (SMP; data not shown).

#### Lamina propria and submucosa

Haematoxylin and eosin staining and c‐kit‐IR labelling revealed the presence of a mild inflammation consisting in clusters of immunity cells beneath the lining epithelium and in the mucosa a significant increase in eosinophilic granulocytes (controls: 51.50 ± 6.652; WRS: 76.50 ± 3.775; OB/WRS: 71.50 ± 6.764, *P* < 0.05) and mast cells (controls: 166 ± 7.371; WRS: 231.3 ± 7.219; OB/WRS: 221.3 ± 4.978, *P* < 0.001), in WRS and OB/WRS rats compared to controls.

**Figure 2:**
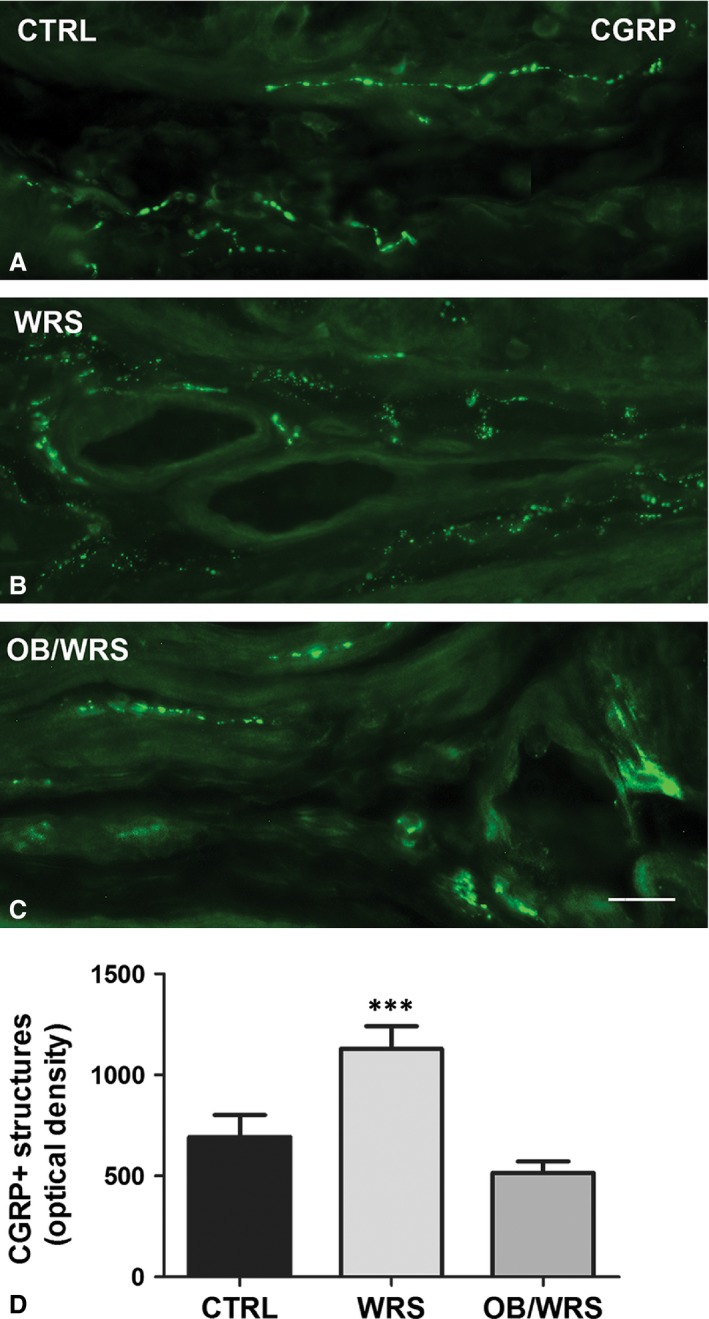
Calcitonin gene‐related peptide (CGRP)‐IR. Varicose axons are numerous within the *muscularis mucosa* and, in the submucosa, close to the wall of blood vessels and at SMP ganglia. In WRS rats (**B**), CGRP‐IR is increased at all levels respect to controls (**A**) and OB/WRS rats (**C**). Bar: **A**–**C** = 25 μm. (**D**) The quantification of all the nerve fibres shows a statistical significant increase in the WRS rats (***P < 0.0001).

CGRP‐IR nerve fibres were significantly increased in WRS rats. This increase was prevented by OB treatment, especially in the submucosa (Fig. 2A–D and Table 1).

**Table 1:** Results of quantitative analysis; *p<0.02, **p<0.001, ***p<0.0001

Neuron number	CTRL	WRS	OB/WRS
NK1r			
MP	41.37 ± 3.547	25.71 ± 2.031*	35.12 ± 2.031
SMP	6.500 ± 0.500	9.500 ± 1.555	7.000 ± 0.577
nNOS			
MP	35.25 ± 2.358	24.06 ± 1.253**	33.94 ± 2.203
SMP	10.00 ± 1.398	3.600 ± 0.921*	6.600 ± 1.024*
VIP			
MP	39.37 ± 0.895	30.44 ± 1.554*	38.25 ± 3.660
SMP	21.50 ± 2.001	15.29 ± 0.905*	19.75 ± 1.090
ChAT			
MP	97.00 ± 4.137	86.87 ± 2.782	74.33 ± 4.621**
SMP	18.73 ± 1.649	17.80 ± 1.868	14.33 ± 1.476
CRF1r			
MP	28.63 ± 4.799	52.64 ± 7.838*	35.07 ± 3.446
CRF2r			
MP	55.30 ± 3.019	77.40 ± 4.290***	78.50 ± 3.349***

Integrated density	CTRL	WRS	OB/WRS
nNOS			
Muscle wall	37,366 ± 207	22,339 ± 134***	36,659 ± 180
SP			
Muscle wall	8079 ± 697	5479 ± 462*	7701 ± 719
CGRP			
Mucosa‐submucosa	693.7 ± 108	1130 ± 111***	515.4 ± 56.07
S100β			
Muscle wall	20,019 ± 188	11,844 ± 167**	20,588 ± 153
Mucosa‐submucosa	6496 ± 525	1879 ± 294***	5961 ± 518

S100β‐IR structures were significantly decreased in WRS rats compared to controls. This decrease was not present in OB/WRS rats (Table 1).

**Figure 3:**
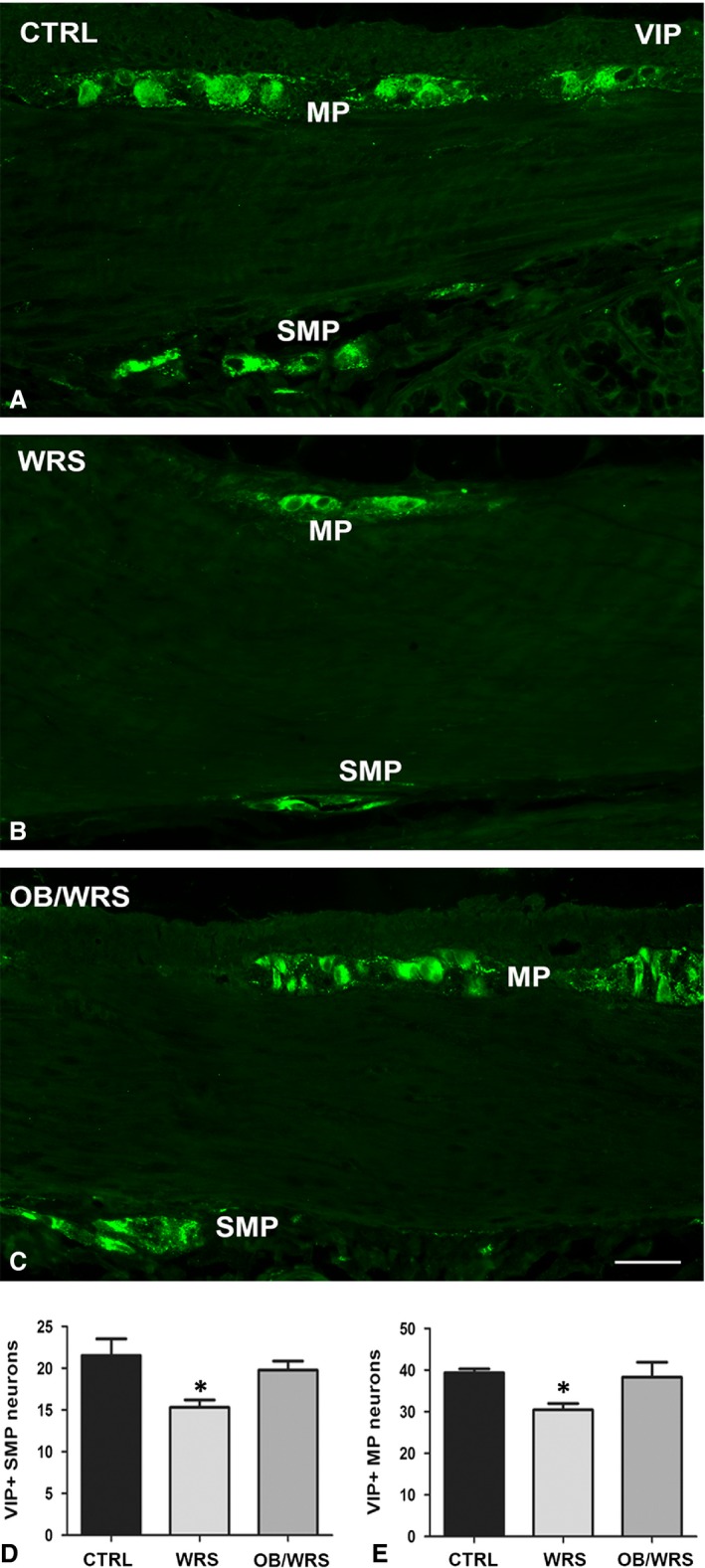
Vasoactive intestinal peptide (VIP)‐IR. Numerous IR neurons are seen at both myenteric (MP) and submucous (SMP) plexuses in all the animals (**A**–**C**); however, in the WRS rats, their number is significantly decreased (**D** and **E**) respect to controls and OB/WRS animals (*P < 0.02). Bar: **A**–**C** = 50 μm.

VIP‐IR neurons were decreased in WRS rats. This decrease was prevented in OB/WRS group (Fig. 3A–D and Table 1).

nNOS‐IR neurons were significantly decreased either in WRS or OB/WRS rats compared to controls (Table 1).

NK1r‐ and ChAT‐IR neuron number was unchanged in the SMP among the groups of animals (Table 1). To note, whereas in controls and OB/WRS rats, the NK1r‐IR labelling was located along the plasma membrane, in WRS rats, it was mainly distributed in the cytoplasm.

#### Muscle wall

**Figure 4:**
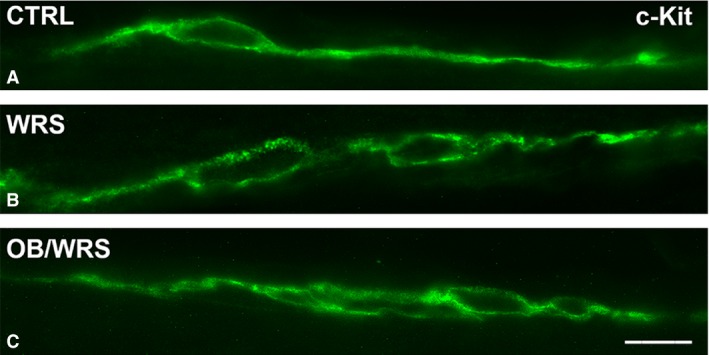
Interstitial cells of Cajal (ICC) identifiable by their c‐kit‐IR. In **A**–**C**, typical ICC, with a spindle shape and a peripheral c‐kit‐positivity, are located at the submucous border of the circular muscle layer. In both WRS (**B**) and OB/WRS (**C**) rats the c‐kit labelling is discontinuous instead to be uniformly distributed, as in controls (**A**), along the entire cell contour. Bar: **A**–**C** = 50 μm.

c‐kit‐IR interstitial cells of Cajal showed no changes in organization, size, shape and number; however, the labelling distribution appeared clustered in OB/WRS and WRS rats instead to be uniformly distributed along the cell profile as in controls (Fig. 4A–C).

**Figure 5:**
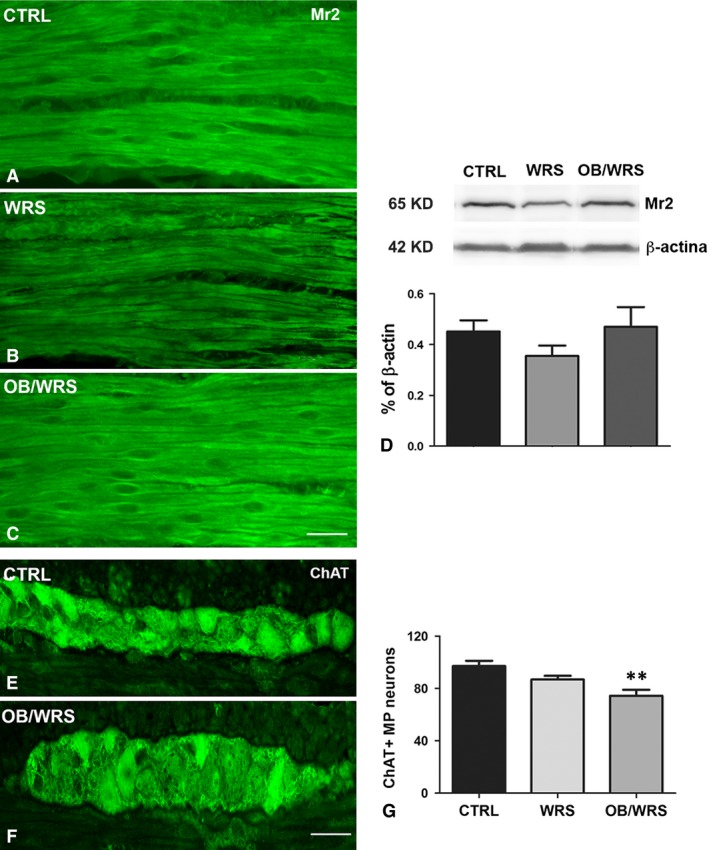
(**A**–**D**) Muscarinic receptor type 2 (Mr2)‐IR. Smooth muscle cells are intensely stained. However, intensity is lower in the WRS (**B**) rats compared to controls (**A**) and OB/WRS (**C**) ones. Bar: **A**–**C** = 50 μm. (**D**) Western blot shows that the Mr2‐IR decrease observed in the WRS rats is not significant. (**E**–**G**) Choline acetyl transferase‐IR (ChAT)‐IR. Numerous IR neurons are seen in all the animals (**E** and **F**). Bar: **E** and **F** = 50 μm. (**G**) The quantification of the myenteric ones shows that in the OB/WRS animals, there is a significant decrease in their number respect to controls and WRS rats (**P < 0.001).

Mr2‐IR in the smooth muscle cells was decreased in WRS rats compared to controls and OB/WRS rats (Fig. 5A–C). Quantization of the receptor by WB confirmed this decrease (Fig. 5D).

ChAT‐IR neuron number showed no change in WRS rats while it was significantly decreased in OB/WRS rats (Fig. 5E–G and Table 1).

S100β‐IR structures were significantly reduced in WRS rats compared to controls; this decrease was prevented by OB treatment (Table 1).

**Figure 6:**
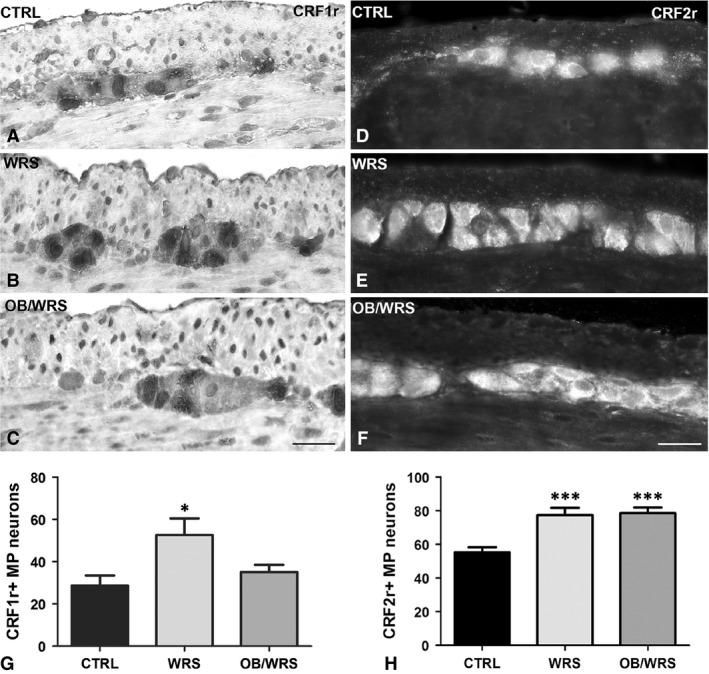
Corticotrophin releasing factor receptor 1 and 2 (CRF1r and CRF2r)‐IR. The labelling is expressed by numerous myenteric neurons in controls (**A** and **D**), WRS (**B** and **E**) and OB/WRS (**C** and **F**) rats. Bar: **A**–**F** = 50 μm. (**G**) The quantification of the CRF1r‐IR neurons shows a statistically significant increase in stressed animals that was prevented in OB treated rats (*P < 0.02). (**H**) The quantification of CRF2r‐IR neurons shows a statistical significant increase in WRS and OB/WRS rats (***P < 0.0001).

CRF1r‐ and CRF2r‐IR neurons (Fig. 6A–F) were significantly increased in WRS rats compared to controls. To note, OB prevented the CRF1r increase exclusively (Fig. 6G and H, Table 1).

**Figure 7:**
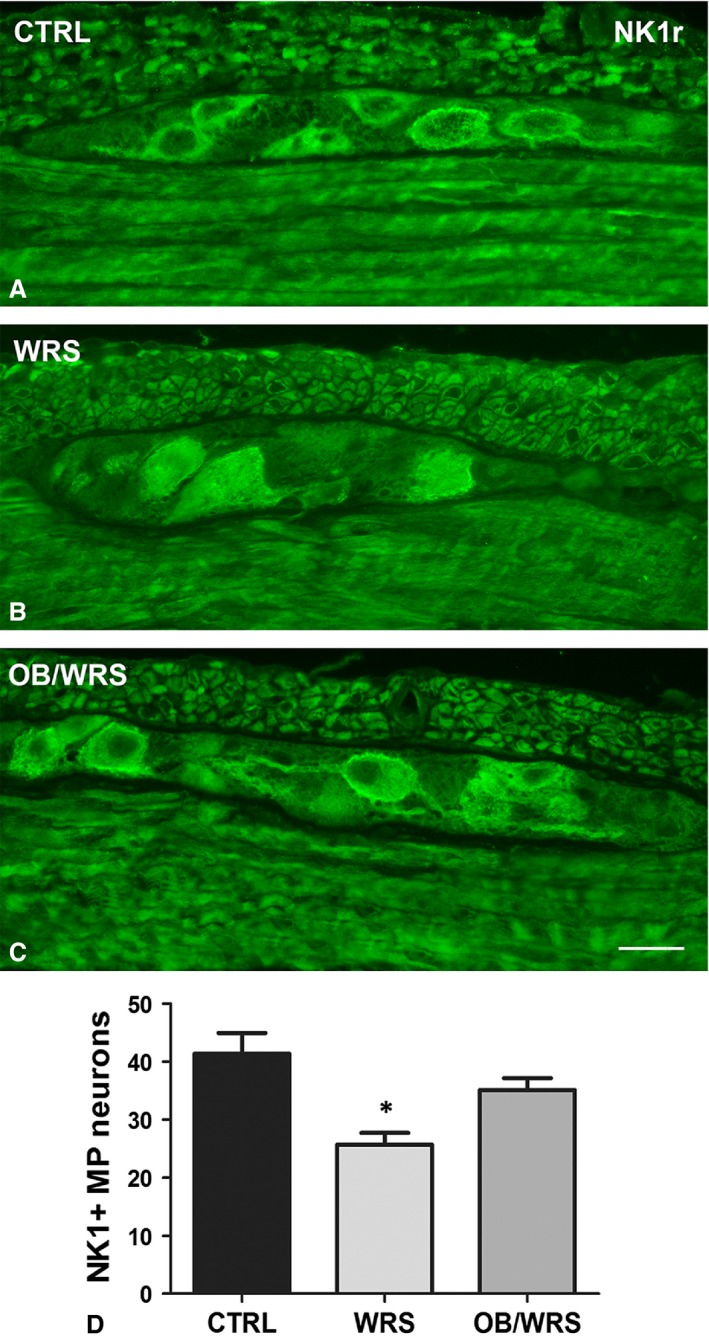
Neurokinin receptor 1 (NK1r)‐IR. In control and OB/WRS rats, the NK1r labelling is mainly located along the neuronal plasma membrane (**A** and **C**) while in the WRS rats (**B**) the majority of the neurons are labelled also in the cytoplasm. MP: myenteric plexus. Bar: **A**–**C** = 25 μm. (**D**) The quantification of the myenteric neurons shows that in the WRS animals, there is a significant decrease in their number respect to controls and OB/WRS rats (*P < 0.02).

VIP‐, nNOS‐ and NK1r‐IR neuron number was significantly decreased in WRS rats; these decreases were completely prevented in OB treated rats (Figs 3A–C, E and 7D; Table 1). Similar to the SMP neurons, in controls and OB/WRS rats the NK1r‐IR labelling was mostly distributed along the plasma membrane (Fig. 7A and C), while in WRS rats the majority of the neurons were labelled also in the cytoplasm (Fig. 7B).

SP‐ and nNOS‐IR nerve structures were significantly decreased in WRS rats; these decreases were undetected in OB/WRS group of animals (Table 1).

## Discussion

This study demonstrates that repeated OB administration in rats subjected to acute WRS is able to counteract most of the morphological changes caused by this psychosocial stressor in the colonic wall. In particular, the drug prevents the decrease in SP‐, NK1r‐, nNOS‐, VIP‐ and S100β‐IR as well as the increase in CGRP‐, and CRF1r‐IR detected in WRS rats. As expected, OB does not interfere with the mild mucosal inflammation because of WRS. Interestingly, OB treatment *per se* increases the Mr2 expression in the muscle wall and decreases the number of the ChAT‐IR myenteric neurons. Noteworthy, OB does not affect the increase in CRF2r‐IR neurons observed in WRS rats. With regard to the functional findings, OB is able to significantly reduce the number of spontaneous AC compared to WRS and control rats but not of those induced by volume distention.

Several studies have been addressed to identify the OB molecular targets 15, 16. The results obtained in *in vitro* experiments after acute exposition of rat colonic strips to the drug showed that OB behaves as antagonist of the L‐type Ca^2+^ channel, M2 and neurokinin receptors 3, 17, 18. These effects were considered the consequence of the OB interaction with the colonic smooth muscle cells because of the very poor systemic absorption of the drug 2. Recently, a series of publications where OB was administered repeatedly has allowed to extend the knowledge on the complexity of OB pharmacology unmasking a constant involvement of the enteric nervous system in mediating the drug actions 6, 7, 8.

Otilonium bromide is commonly used to control the main symptoms of IBS, a chronic disease characterized by visceral pain and/or discomfort, hypersensitivity and abnormal motor responses 19. Although the aetiopathogenesis of IBS is only partially understood, a main role has been attributed to psychosocial stressors of different origin 20, 21. The WRS is considered a psychosocial stress model adequate to reproduce in the animals, some of the typical symptoms and signs of IBS 22, 23, 24. Accordingly, we recently reported functional and morphological alterations in WRS rat similar to those described in IBS patients 12 and, presently, we demonstrate that repeated OB administration prevents most of the changes caused by the WRS, particularly those on the enteric nervous system. However, the cellular mechanism through which OB could interfere with the nerve structures is undefined. Among the several pharmacological properties attributed to OB, the inhibition of the calcium entry by binding to the L‐type Ca^2+^ channels has been considered the most significant 3, 6, 16, 17. In this respect, since the neurons express the L‐type Ca^2+^ channels, the possibility that OB, blocking these channels, could modify the neuronal response to physiological 6, 7 or pathological 12 stimuli has to be considered. As a matter of fact the variations in the neurotransmitters’ expression found in the myenteric neurons after repeated OB treatment 6, 7, 8 are coupled to significant changes in neuronal activity 8.

Of particular interest are the changes in the CRF1r and CRF2r expression presently detected in the myenteric neurons of WRS rats and in WRS rats pre‐treated with OB. The presence of these two receptors was already demonstrated either by PCR techniques in the human colon 13 or by immunohistochemistry in the rat colon 14 and it was shown that the CRF2r is higher expressed compared to the CRF1r. In agreement, we presently detect a greater number of CRF2r‐IR neurons compared to the CRF1r‐IR ones. Pharmacological studies have shown that the endogenous ligand cortical releasing factor (CRF) shows higher affinity to the CRF1r respect to the CRF2r 14, 25. Cortical releasing factor is considered the primary hypothalamic mediator of the mammalian neuroendocrine and behavioural responses to stress 26 and the adverse gastrointestinal effects (visceral hypersensitivity, increased colonic transit) caused by its injection or by psychosocial stressors are blocked by CRF1r antagonists 27, 28. With regard to the CRF2r role, it was reported that injection of selective agonists did not provoke any measurable response in the rat colonic wall 14 and it has been suggested that the CRF2r activation could negatively modulate the CRF1r mediated responses. In fact, the administration of the selective CRF2r agonist, Urocortin 2, inhibits the CRF1r mediated stimulation of colonic motor function induced by acute partial restrain stress or by CRF injection 14. In a recent paper, we reported that WRS causes an increase in the CRF1r expressing neurons in the myenteric plexus of rat colon. Presently, we show also a significant increase in the CRF2r‐IR myenteric neurons in WRS rats. Thus, it can be inferred that stress causes a complex neuronal response consisting not only of a predictable increase in CRF1r, as mediator for the adverse local effects, but also of an unexpected increase in CRF2r. On the basis of the literature, this latter finding might be interpreted as an attempt to cope to the CRF1r mediated stress effects that could bring to an overdrive state and predispose to diseases. The relevance of the CFR2r in determining the outcome of a stress condition is likely underestimate. In a recent report on the lack of a CRF1r antagonist efficacy to ameliorate colonic symptoms in IBS patients 29, it has been suggested that impairment in the CRF1r‐CRF2r integrated response might be implicated in the pathogenesis of this disease.

In the muscle wall of WRS rats, the expression of the inhibitory (nNOS, VIP) and excitatory (SP/NK1r) neurotransmitters investigated is decreased suggesting that stress causes an impairment of the neuronal activity. Reasonably, this impairment is triggered by the CRF through its receptors. To note, the CRF, beyond the central nervous system, is also expressed and likely released by the enteric neurons and, in stress conditions, the number of the neurons expressing the CRF is increased 13. Few and partial information is available on the specific neuronal population releasing the CRF and/or expressing the CRF receptors 30, 31. Noteworthy, presently, we show that repeated administration of OB prevents almost of the neurotransmitters’ decreases and the overexpression of the CRF1r in the myenteric plexus without modifying that of the CRF2r. Our experimental conditions do not allow to establish a direct relationship between the two events; however, the ability of OB to prevent the neurotransmitters’ changes due to stress and the reduction in the excitatory ChAT expressing neurons, caused by the drug *per se,* might explain the spasmolytic activity exerted by OB and the relief from most of the IBS symptoms.

As expected OB does not show any anti‐inflammatory activity when measured as cell infiltrate and mast cell increase. However, through its ability to interact with the nerve structures, OB prevents the increase in the CGRP expression in the submucosa and mucosa of WRS rats. CGRP is mainly expressed by sensory neurons and in IBS patients or in rats underwent to maternal deprivation, CGRP‐positive fibres are increased and strictly associated with mast cells 32, 33, 34. Moreover, stress conditions favour mast cell degranulation and release of inflammatory mediators able to sensitize sensory afferents such as those carrying CGRP 35, 36. Furthermore, the rectal biopsy samples taken from patients with well‐characterized rectal hypersensitivity showed a marked increase in submucosal CGRP 11. Therefore, although OB does not interfere with the mast cell increase present in WRS rats, it is reasonable to infer that, because of its ability to prevent the overexpression of CGRP, it might also ameliorate symptoms such as pain.

The results obtained with the functional studies appear contrasting also in view of the known OB efficacy in counteract visceral sensitivity in IBS patients 37, 38. Interestingly, OB causes a significant reduction in the spontaneous AC contraction respect to controls and WRS rats. This datum indicates that OB induces muscle relaxation, thus reducing the visceral motility. The inability of OB to reduce the number of AC because of volume distention might be explained by the modalities provided by the CRD test. In fact, it has been reported that the application of a progressive volume of distention increasing the painful sensations recruits central efferent fibres 22 insensitive to OB effects 3. Accordingly, when this test was applied to measure the efficacy of other drugs known to be able to reduce the visceral sensitivity in humans, the results failed 39.

In conclusion, this study confirms the WRS as a model suitable to mimic most of the IBS symptomatology and adequate to investigate the OB cellular targets to ameliorate our understanding of the drug mechanisms of action in IBS. The findings obtained indicate that most of the OB activity is exerted at the enteric nervous system level by preventing the neuronal changes caused by the stress. How OB act at the cellular level is not defined yet; however, its ability to block the calcium entry could represent a plausible explanation. This pharmacological property might play a role also in avoiding the increase in the neuronal cells that express the CRF1r, as observed in the WRS rats. We are aware that the combination of a repeatedly pre‐treatment with the application of an acute model of stress might represent a limitation of this study. However, the results obtained in the clinical studies of OB efficacy in IBS patients 40 have brought to hypothesize that cyclic or intermittent treatment, independently on the presence of the IBS symptoms, might be an adequate therapeutic strategy to manage the IBS 41.

## Conflict of interest

We declare that no conflict of interests in our study is going to disclose.

## Author contributions

CT performed the immunohistochemical and biomolecular research; VG performed the WRS and functional experiments; CT and VG analysed the data and performed the statistics; SE performed a critical review of the manuscript and contributed to the support of the study; MSFP prepared the figures and performed a critical revision of the manuscript; CT, MSFP and MGV wrote the article; MGV carried out a critical revision of the data obtained, a critical review of manuscript, study concept and design for important intellectual content and obtained funding.

## Supporting information


**Table S1** Detailed description of treatment and protocol applied for each experimental group during the first and second phase.

**Table S2** List of primary antibodies.

**Data S1** Materials and methods.
