# Supplementary material for: Repeated otilonium bromide administration prevents neurotransmitter changes in colon of rats underwent to wrap restraint stress
Source: J Cell Mol Med. 2016 Nov 20;21(4):735–45. doi: 10.1111/jcmm.13016 (PMC5345670; doi:10.1111/jcmm.13016)
Supplement: Supplementary file 1 — Table S1 Detailed description of treatment and protocol applied for each experimental group during the first and second phase. [file JCMM-21-735-s001.docx]

|  | ***1^st^ phase*** | | | | | | | | | | |
| --- | --- | --- | --- | --- | --- | --- | --- | --- | --- | --- | --- |
|  |  | | | **Treatment** | | | | |  | | |
| **Group** | **n** | **Drug** | | **Dose/volume** | | **Route** | **Schedule** | | **Sensitization** | | **Protocol** |
| CTRL | 8 | s.w | | | n.a./5ml/kg | *p.o.* | Once on D_0_  4 hs before  CRD test. | | None | | CRD test and  fecal pellet collection |
| WRS | 8 | s.w | | | n.a./5ml/kg |  |  |  | WRS | |  |
| OB/WRS | 8 | OB | | | 2mg/kg/5ml/kg |  |  |  | WRS | |  |
| OB/WRS | 8 | OB | | | 20mg/kg/5ml/kg |  |  |  | WRS | |  |
|  |  | | | | | | | | | | |
|  | ***2^nd^ phase*** | | | | | | | | | | |
|  |  | | | | **Treatment** | | |  | | | |
| **Group** | **n** | | **Drug** | | **Dose/volume** | **Route** | **Schedule** | **Sensitization** | | **Protocol** | |
| CTRL | 8 | | s.w | | n.a./5ml/kg | *p.o.* | Twice daily  from D_-10_ to D_0_ and 4 hs before CRD test. | None | | CRD test and  fecal pellet collection | |
| CTRL | 5 | | s.w | | n.a./5ml/kg |  |  | None | | Colon removal | |
| WRS | 8 | | s.w | | n.a./5ml/kg |  |  | WRS | | CRD test and  fecal pellet collection | |
| WRS | 5 | | s.w | | n.a./5ml/kg |  |  | WRS | | Colon removal | |
| OB/WRS | 8 | | OB | | 20mg/kg/5ml/kg |  |  | WRS | | CRD test and  fecal pellet collection | |
| OB/WRS | 5 | | OB | | 20mg/kg/5ml/kg |  |  | WRS | | Colon removal | |

**Table 1.** Detailed description of treatment and protocol applied for each experimental group during the 1^st^

and 2^nd^ phase. n: number of rats for experimental group; s.w.: sterile water; n.a.: not applicable; D: day; *p.o.:* per os.
