# Supplementary material for: Repeated otilonium bromide administration prevents neurotransmitter changes in colon of rats underwent to wrap restraint stress
Source: J Cell Mol Med. 2016 Nov 20;21(4):735–45. doi: 10.1111/jcmm.13016 (PMC5345670; doi:10.1111/jcmm.13016)
Supplement: Supplementary file 2 — Table S2 List of primary antibodies. [file JCMM-21-735-s002.docx]

**Table 2. List of primary antibodies**

| **Primary antibody** | **Host** | **IHC WB** | **Producer** |
| --- | --- | --- | --- |
| Anti-Substance P | Guinea Pig | 1:500 | Abcam, Cambridge, UK |
| Anti-NK1r | Rabbit | 1:200 | Chemicon Temecula, CA, USA |
| Anti-nNOS | Rabbit | 1:1500 | Millipore, Bedford, MA, USA |
| Anti-ChAT | Goat | 1:50 | Millipore, Bedford, MA, USA |
| Anti-Mr2 | Rabbit | 1:50 1:500 | Alomone Labs. Jerusalem, Israel |
| Anti-PGP9.5 | Rabbit | 1:400 | AbDSerotec, Kidlington, UK |
| Anti-c-Kit | Rabbit | 1.300 | Dako, Glostrup, DK |
| Anti-S100β | Mouse | 1:200 | Abcam, Cambridge, UK |
| Anti-CGRP | Rabbit | 1:400 | Abcam, Cambridge, UK |
| Anti-VIP | Rabbit | 1:50 | Santa Cruz Biotech., Santa Cruz, CA, USA |
| Anti-CRF1r | Goat | 1:50 | Santa Cruz Biotech., Santa Cruz, CA, USA |
| Anti-CRF2r | Goat | 1:200 | Santa Cruz Biotech., Santa Cruz, CA, USA |
