# Supplementary material for: Repeated otilonium bromide administration prevents neurotransmitter changes in colon of rats underwent to wrap restraint stress
Source: J Cell Mol Med. 2016 Nov 20;21(4):735–45. doi: 10.1111/jcmm.13016 (PMC5345670; doi:10.1111/jcmm.13016)
Supplement: Supplementary file 3 — Data S1 Materials and methods. [file JCMM-21-735-s003.docx]

**Supplementary Materials and Methods**

**Wrap Restraint Stress (WRS)**

Stress was induced using a wrap partial restraint stress procedure, an acute non-ulcerogenic model of restrain. The stress session was performed between 10 am and 12 am, in order to minimize the influence of circadian rhythms. Animals were lightly anesthetized with isofluorane and then their upper forelimbs and thoracic trunk were wrapped using adhesive tape for 2 h from T_0-2h30_ to T_0-30_ (Fig. 1). Control animals were lightly anesthetized with isofluorane, but were not wrapped.

**Fecal pellet collection**

Fecal pellets were collected from T_0-2h30_ to T_0-30_, during the stress application. At the end of the collection period, the number of fecal pellets and their total weight were determined.

**Colorectal distension (CRD) and abdominal contraction (AC) recordings**

At the end of the WRS procedure, rats were allowed to recover from T_0-30_ to T_0_. Then, from T_0_ to T_0+30,_ the animals were placed in a plexiglas tunnel and a distension balloon, mounted on a catheter filled with water and connected to a pressure transducer, was introduced into the colorectum for CRD and AC recordings. In order to minimize stress reaction that could be associated with this procedure, all the animals were acclimated to the tunnel a few days before distension. To allow CRD and AC recordings, the pressure transducer was connected to both a syringe and an amplifier (TBM4M, World Precision Instruments, Hitchin, UK). Once amplified, the signal was processed using a data acquisition unit (Powerlab, AD Instruments, Oxford, UK) and then displayed and recorded on a personal computer using dedicated software (Chart, AD Instruments, UK). From T_0_, the balloon was progressively inflated step by step from 0 to 0.4, 0.8 and 1.2 ml. For each animal, the number of AC produced during each step of distension lasting 5 min was recorded.

**Western blot**

Full-thickness samples of ascending colon were quickly minced and homogenized with a tissue homogenizer (Ing. Terzano, Milan, Italy) in a cold lysis buffer composed of: 10 mM Tris/HCl pH 7.4, 10 mM NaCl, 1.5 mM MgCl2, 2 mM Na2EDTA, 1 mM phenylmethylsulfonyl fluoride (PMSF), 1% Triton X-100, added with 1X Sigmafast Protease Inhibitor cocktail tables (Sigma-Aldrich). Upon centrifugation at 13000 g for 30 min at 4°C, the supernatants were collected, and the total protein content was measured spectrophotometrically using a micro BCA Protein Assay Reagent Kit (Thermo scientific). Samples (70 μg of proteins per well) and appropriate molecular-weight markers (Bio-Rad, Hercules, CA, USA) were loaded onto a 7.6% SDS-PAGE gel and resolved by standard electrophoresis. The proteins were then blotted (150 V, 1 h) onto polyvinylidene fluoride (PVDF) microporous membrane (Millipore Corporation, Bedford, MA, USA). After washings in PBS 0.1% Tween-20 (PBS-T, Sigma-Aldrich), the membranes were treated with a blocking buffer made by 5% no fat dry milk (Sigma-Aldrich) diluted in PBS-T for 1 h at RT, then incubated with the anti-M2r primary antibody ON at 4°C while being stirred. The immunoreaction products were revealed by incubating membranes with appropriated peroxidase conjugated secondary antibody (goat anti-rabbit; 1:15000, Jackson ImmunoResearch, West Grove, PA, USA) for 1 h at RT. Immunoreactivity was revealed by an enhanced chemiluminescence reagent (Immune-StarTM HRP Chemiluminescent Kit, Bio RadHercules, CA, USA) and was captured using ImageQuant 350 Imager (GE Healthcare, Buckinghamshire, UK). In order to normalize the values of the antibody, all western blot runs were stripped (Stripping buffer, Thermo scientific) and then immunostained with anti-β-actin (1:20000; Sigma-Aldrich) assuming actin as the control housekeeping protein.
